# Mapping pleiotropic loci using a fast-sequential testing algorithm

**DOI:** 10.1038/s41431-021-00911-z

**Published:** 2021-06-18

**Authors:** Fernando M. Aguate, Ana I. Vazquez, Tony R. Merriman, Gustavo de los Campos

**Affiliations:** 1 Department of Epidemiology & Biostatistics, IQ – Institute for Quantitative Health Science and Engineering, Michigan State University, East Lansing, MI USA; 2 Department of Medicine, University of Alabama at Birmingham, Birmingham, AL USA; 3 Department of Statistics & Probability, Michigan State University, East Lansing, MI USA

**Keywords:** Computational biology and bioinformatics, Genome informatics

## Abstract

Pleiotropy (i.e., genes with effects on multiple traits) leads to genetic correlations between traits and contributes to the development of many syndromes. Identifying variants with pleiotropic effects on multiple health-related traits can improve the biological understanding of gene action and disease etiology, and can help to advance disease-risk prediction. Sequential testing is a powerful approach for mapping genes with pleiotropic effects. However, the existing methods and the available software do not scale to analyses involving millions of SNPs and large datasets. This has limited the adoption of sequential testing for pleiotropy mapping at large scale. In this study, we present a sequential test and software that can be used to test pleiotropy in large systems of traits with biobank-sized data. Using simulations, we show that the methods implemented in the software are powerful and have adequate type-I error rate control. To demonstrate the use of the methods and software, we present a whole-genome scan in search of loci with pleiotropic effects on seven traits related to metabolic syndrome (MetS) using UK-Biobank data (n~300 K distantly related white European participants). We found abundant pleiotropy and report 170, 44, and 18 genomic regions harboring SNPs with pleiotropic effects in at least two, three, and four of the seven traits, respectively. We validate our results using previous studies documented in the GWAS-catalog and using data from GTEx. Our results confirm previously reported loci and lead to several novel discoveries that link MetS-related traits through plausible biological pathways.

## Introduction

Many human diseases (e.g., hypertension, gout, and diabetes) cluster into syndromes. Evidence from quantitative genetic studies [1, 2] and from genome-wide association (GWA) analyses [3] suggest that pleiotropy (i.e., variants with simultaneous effects on several traits) is an important driver of the genetic correlation between comorbid conditions. Therefore, in recent years, there has been an increased interest in mapping genetic loci with pleiotropic effects [4–6]. However, mapping these loci on multiple traits can be computationally and statistically challenging because of the large number of tests involved. For example, in a system of three traits, there are four possible configurations involving pleiotropy: one for associations with three traits and three involving two traits. The number of possible pleiotropic configurations grows exponentially with the number of traits; for example, in a system of 10 traits there are 1013 configurations which involve the same variant having effects in at least two traits. This exponential growth in the number of hypotheses that need to be tested creates obvious computational difficulties, makes type I error control challenging, and renders interpretation and communication of results difficult. These challenges get exacerbated by the fact that whole-genome scans require performing these tests for potentially millions of variants and by the very large sample size of modern biobanks.

To confront the challenges posed by the analysis of systems of many traits, some authors considered using phenotype-derived principal components (PCs) as traits in GWA analyses. For example, this approach has been used to identify variants associated with patterns shared across multiple MetS-related traits [7]; however, this approach has several limitations. Firstly, the phenotype-derived PCs often lack a clear biological interpretation, which can lead to difficult-to-interpret GWA results. Secondly, PCs are derived from phenotypic (co)variance patterns, which may be highly influenced by shared environmental factors, thus reducing the power to detect genetic associations [8].

Schaid et al. [9] proposed a formal test for pleiotropy that addresses many of the statistical challenges posed by GWA analyses of systems involving many traits. The approach uses a multivariate model and a sequence of likelihood-ratio tests (sLRT) designed to identify the set of SNPs that are associated with at least two, three, …, *p* traits in the system. This sequential test controls the type-I error rate while offering the power of multi-trait models. The methodology developed by Schaid et al. has several attractive features. Firstly, the test uses all the information available, without relying on dimension reduction techniques such as PCs. Secondly, the approach groups the possible pleiotropic configurations into a few meaningful states where the locus affects at least *q* trait(s) in the system (*q* = 1,…, *p*), thus facilitating interpretability. Finally, the *p* values for each of these states are derived using a well-established intersection-union test that guarantees adequate type I error control, regardless of the complexity of the system [10]. However, the sLRT is computationally demanding, and the existing software [11] that implements it does not scale to whole-genome scans involving many traits and large sample size.

Therefore, in this study, we developed an approximation to the sLRT that achieves the same power and error-control performance but is orders of magnitude faster to compute. Our approach (pleiotest) succeeds at evaluating the sequential test faster by using three main strategies: (i) instead of using a likelihood ratio test with decorrelated data, we use Wald’s test [12] with a simplified version of the variance of the coefficients, which is slightly faster to compute. (ii) Following Turley et al. [13], we approximate the error (co)variance matrix with an estimate derived from covariate (e.g., sex, age, evaluation center, etc.) adjusted phenotypes. For complex traits, this (co)variance matrix approximates well the residual (co)variance matrix because individual SNPs explain only a small fraction of the phenotypes. Finally, (iii) we implemented the core of the computations using the C++ language and integrated this into an R package that is both fast and user friendly. Importantly, our package is compatible with the BEDMatrix R-package [14], which implements memory mapping for binary genotype files in bed format; thus, enabling big data analysis within the R-environment.

In this study we describe the methodology implemented in pleiotest, present extensive simulations that show that the proposed approximation has the same power and error control than the original test, and provide a benchmark that shows that our method is orders of magnitude faster than the sLRT and scales well to analyses involving many traits (e.g., 10 or more) and very large sample sizes (e.g., *n* > 300 K; *K* = 1000). Finally, to demonstrate the use of pleiotest to map pleiotropic loci with big data, we applied the sequential testing to seven traits related to metabolic syndrome (MetS), using a dataset of distantly related white-European individuals from the UK-Biobank (*n* = ~316 K and ~624 K variants).

## Materials and methods

### Statistical methods

Consider a single-marker regression (SMR) in which a (centered and covariate-adjusted) phenotype (***y***) is regressed on an SNP genotype (***x***) using a linear model of the form

$${\boldsymbol{y}}_j = {\boldsymbol{x}}\beta _j + \varepsilon _j$$

where ${\boldsymbol{y}}_j = \left( {y_{j1}, \ldots ,y_{jn}} \right)^\prime$ is the vector of phenotypes for the *j*th trait (*j =* 1*,..., p*), ${\boldsymbol{x}} = \left( {x_1, \ldots ,x_n} \right)^\prime$ is a vector containing the (mean-centered) genotypes of each of the individuals at a given SNP, *β*_*j*_ is the SNP effect on the *j*th trait, and $\varepsilon _j = \left( {\varepsilon _{j1}, \ldots ,\varepsilon _{jn}} \right)^\prime$ is the vector of error terms for the *j*th trait which we will assume to be normally distributed (more below).

For the balanced case (i.e., when all subjects had data for all traits) a multi-trait SMR can be expressed using:

$${\boldsymbol{y}} = {\boldsymbol{X\beta }} + \varepsilon$$

where ${\boldsymbol{y}} = ({{\boldsymbol{y}}^\prime_1,{\boldsymbol{y}}^{\prime}_2, \ldots ,{\boldsymbol{y}}^{\prime}_{\it{p}}})^\prime$ is a vector containing phenotype values for each individual and trait, ${\boldsymbol{X}} = {\boldsymbol{I}}_{\boldsymbol{p}} \otimes {\boldsymbol{x}}$, ${\boldsymbol{\beta }} = \left( {\beta _1, \ldots ,\beta _p} \right)^\prime$ is a vector containing the effects of the SNP on each of the *p*-traits, and $\varepsilon = ({\varepsilon}^{\prime}_1,\varepsilon ^{\prime}_2, \ldots ,\varepsilon ^{\prime}_p)^\prime$ is a vector of error terms, assumed to follow a Multivariate Normal Distribution (*MVN*) with zero mean and (co)variance matrix ***R***⊗***I***_*n*_. Here, ⊗ represents the Kronecker product operator, ***I***_*n*_ stands for an n-dimensional identity matrix, and **R** is a (*p*×*p)* within-subject (co)variance matrix of the error terms.

For the balanced case, the Maximum Likelihood Estimator of the SNP effects (also known as Seemingly Unrelated Regressions [15], SUR, estimator) is

1 $${\hat{\boldsymbol{\beta }}} = \left[ {{\boldsymbol{X}}^\prime {\mathbf{\Omega }}^{ - 1}{\boldsymbol{X}}} \right]^{ - 1}{\boldsymbol{X}}^\prime {\mathbf{\Omega }}^{ - 1}{\boldsymbol{y}}$$

where ${\mathbf{\Omega }}^{ - 1} = {\boldsymbol{R}}^{ - 1} \otimes {\boldsymbol{I}}_n$. The sampling (co)variance matrix of the estimated effects is

2 $$cov\left( {{\hat{\boldsymbol{\beta }}}} \right) = \left[ {{\boldsymbol{X}}^\prime {\mathbf{\Omega }}^{ - 1}{\boldsymbol{X}}} \right]^{ - 1} = {\boldsymbol{R}}/({\boldsymbol{x}}^\prime {\boldsymbol{x}})$$

The (co)variance matrix ***R*** is unknown; therefore, estimates are often obtained using a two-stage procedure where in the first step, each trait is regressed on the SNP separately and the residuals from these single-trait regressions are used to estimate ***R*** using a method of moments estimator

3 $${\hat{\boldsymbol{R}}} = \left\{ \hat R_{jj^\prime} = \left( {n - 1} \right)^{ - 1}\hat \varepsilon ^{\prime}_j\hat \varepsilon _{j^{\prime}} \right\}$$

In the second step, expressions (1) and (2) are evaluated using ${\hat{\boldsymbol{R}}}$ in place of ***R***. This procedure requires fitting models twice for each SNP, which is computationally demanding.

For most complex traits, individual SNPs typically explain a small fraction of the variance of phenotypes (e.g., loci with large effects may explain up to 3% of the trait variance). Therefore, following Turley et al. [13], we propose using mean-centered and covariate-adjusted phenotypes (***y***_*j*_) instead of single-marker regression residuals ($\hat \varepsilon _j$) in (3): ${\boldsymbol{S}} = \{ S_{jj^{\prime}} = \left( {n - 1} \right)^{ - 1}{\boldsymbol{y}}^{\prime}_j{\boldsymbol{y}}_{j^\prime }\}$. Since the adjusted phenotypes do not depend on SNP genotypes, expression (3) needs to be computed only once, using adjusted phenotypes instead of model residuals; thus, avoiding the need to fit regressions twice for every SNP. Since ${\boldsymbol{S}} - {\hat{\boldsymbol{R}}}$ is guaranteed to be positive semi-definite, using ***S*** in place of ***R*** can only lead to slightly conservative inferences. Although this could reduce power, this would only happen if an individual SNPs had a sizable effect (i.e., when${\boldsymbol{S}} \gg {\hat{\boldsymbol{R}}}$); however, in that case power is expected to be very high even with a small sample size (e.g., *n* = 3000). Therefore, the proposed approximation is expected to preserve error control and power – our simulation results confirm this expectation.

### Wald test

Schaid et al. [9] proposed testing pleiotropy using a sequential likelihood ratio test (sLRT). For computational convenience, we consider using a sequential Wald’s test with a simplified version of $\left[ {{\boldsymbol{X}}^\prime {\mathbf{\Omega }}^{ - 1}{\boldsymbol{X}}} \right]^{ - 1}$ and ${\boldsymbol{X}}^\prime {\mathbf{\Omega }}^{ - 1}{\boldsymbol{y}}$ that can be obtained even in the unbalanced case (more in Section III of the Appendix). It can be shown that these two tests are equivalent in special cases (e.g., in linear models with balanced data and when variance components are assumed to be known) and, more generally, become equivalent for relatively modest sample sizes [16] (see Section I of the Appendix for further details).

### Sequential testing

Implementing a sequential test for pleiotropy requires evaluating multiple configurations in which SNPs may have effects on 2, 3, …, *p* traits. All the hypotheses that need to be tested can be expressed in linear form $H_0:{\boldsymbol{C\beta }} = 0$. The Wald’s statistic for this linear hypothesis takes the form$w = {\hat{\boldsymbol{\beta}}} ^{\prime}C^{\prime}[{{\boldsymbol{C}}}cov({{\hat{\boldsymbol{\beta}}}}){\boldsymbol{C}}^{\prime}]^{ - 1}{\boldsymbol{C}}\hat {\boldsymbol{\beta}}$. Under the null hypothesis, the test statistic follows a chi-square distribution with degrees of freedom equal to the rank of ***C***. The first step in the sequence test whether the SNP has an effect on at least one trait ($H_0:{\boldsymbol{\beta }} = 0$), thus $w_0 = {\hat{\boldsymbol{\beta}}}^\prime cov({\hat{\boldsymbol{\beta}}})^{ - 1}{\hat{\boldsymbol{\beta}}}\sim$ $\chi _p^2$. If this test is rejected, the next step involves testing whether the SNP has an effect on at least two traits. Here, there is a multiplicity of null hypotheses. The *p* value for the test that the SNP has a non-zero effect in at least two traits is obtained using an intersection-union (IU) test [10]: the *p* values for all possible nulls are computed and the final *p* value is the one corresponding to the minimum Wald’s statistic:$\mathop {{\min }}\limits_{k = 1,..,p} w_k$, where *w*_*k*_ is $w_k = {\boldsymbol{C}}_{\boldsymbol{k}}^\prime {\hat{\boldsymbol{\beta}}}^\prime ({\boldsymbol{C}}_{\boldsymbol{k}}^\prime cov( {{\hat{\boldsymbol{\beta}}}}){\boldsymbol{C}}_{\boldsymbol{k}})^{ - 1}{\boldsymbol{C}}_{\boldsymbol{k}}^\prime {\hat{\boldsymbol{\beta}}}$ . The contrasts needed to obtain the Wald’s statistics for this second step (***C***_*k*_) can be obtained by removing the *k*th row (*k* = 1, 2, …, *p*) of a *p*-dimensional identity matrix (see Section II of the Appendix for an example). If this test is rejected, the following steps in the sequence test whether the SNP has effects on at least 3 traits, then 4 traits, and so on.

### Unbalanced data

Above, we described the implementation of SUR and Wald sequential tests for the case of balanced data. The same tests can be extended to cases involving incomplete phenotype data. We designed an efficient approach for obtaining GLS estimates, SEs and *p* values for the sequential test for the case of unbalanced data. We identify all missing-value patterns present in the data, then we compute the relevant summary statistics for each group, and finally we combine them to obtain the GLS estimates–further details are presented in Section III of the Appendix. Our approach assumes that the missing values are non-informative. Under these conditions, it can be shown that the resulting GLS estimator is unbiased because it is a weighted average of group-specific estimators which are themselves unbiased [17]. However, when the missing values are informative (e.g., when the probability of missing value depends on other trait values or on covariates) the estimator won’t be unbiased unless the missing value process is modeled adequately.

### Software

We implemented the methods described in the preceding sections into the R-package *pleiotest*. The computationally demanding steps of the algorithm are implemented in C++ language, embedded into R-code using the *RcppArmadillo* package. Our package offers functions to perform multi-trait GWA analyses, a sequential test for pleiotropy, and graphical display of pleiotropy analysis. The package and documentation, including standard pipelines and examples can be found in the GitHub repository https://github.com/FerAguate/pleiotest and in The Comprehensive R Archive Network https://cran.r-project.org/web/packages/pleiotest.

### Monte Carlo study

We performed simulations to assess the type I error rate and power of the sequential testing based on Wald’s test with the proposed approximation of the (co)variance matrix (${\boldsymbol{S}} \approx {\hat{\boldsymbol{R}}}$) (hereafter named pleiotest), and those of Schaid’s sLRT. To study type I error rate, we first simulated three traits (*p* = 3; *j =* 1*,..., p*; $y_{ji} = \mu _j + x_i\beta _j + \varepsilon _{ji}$), with $cor\left( {\varepsilon _{ji}} \right)$ = 0.2 or 0.8 and sample size of 3000 or 10,000. Genotypic variants (*x*_*i*_) were simulated from a Binomial distribution with allele frequencies sampled the empirical distribution of minor-allele frequencies ranging from 0.01 to 0.5. In a first setting, there were no genetic effects on any trait ($\beta _1 = \beta _2 = \beta _3 = 0$); subsequently, we simulated a system with the SNP having an effect on trait one only ($\beta _1 \, \ne \, 0;\beta _2 = \beta _3 = 0$). For the trait with a genetic effect, the SNP explained 1% of the phenotypic variance. Clearly, none of these settings involve pleiotropy. We carried out 500 million Monte Carlo (MC) simulations for the scenario with a sample size of 3000 and 100 million for the sample size of 10,000. In the first setting ($\beta _1 = \beta _2 = \beta _3 = 0$) we tested the alternative hypothesis that the SNP had an effect on at least one of the traits (i.e., Ha_1_: $\beta _1 \, \ne \, 0,\beta _2 \, \ne \, 0\;{\mathrm{or}}\;\beta _3 \, \ne \, 0$). In the second setting ($\beta _1 \, \ne \, 0;\beta _2 = \beta _3 = 0$) we tested two-traits pleiotropy (i.e., Ha_2_: $\beta _1 \, \ne \, 0\& \beta _2 \, \ne \, 0$ or $\beta _1 \, \ne \, 0\& \beta _3 \, \ne \, 0$ or $\beta _2 \, \ne \, 0\& \beta _3 \, \ne \, 0$) against the composite null where all *β*_*j*_ are equal to zero or one is different than zero while the others remain equal to zero.

To quantify power, we performed an MC simulation of the three traits simultaneously affected by the same locus. We considered different settings regarding absolute and relative effect sizes. The proportion of variance explained by the locus on trait one varied from 0 (no effect) to 1%. For each setting we considered: (i) equal effect size across traits ($\beta _1 = \beta _2 = \beta _3$; i.e., effect-ratio = 1), and (ii) *β*_1_ twice as big as *β*_2_ and *β*_3_ (effect-ratio = 0.5). We also considered scenarios with homogeneous error correlations of 0.2, 0.5, or 0.8 and sample sizes of 3000, 5000, or 10,000. Combining these scenarios led to 360 (2 × 20 × 3 × 3) different analysis settings. For power analysis (since the Monte Carlo Error is expected to be much lower than for Type-I error rate estimations) we conducted 100,000 MC replicates per setting.

### Computational performance

We measured the computation time that takes for pleiotest to prepare the data (pre-processing), fit the model, and perform the sequential test for pleiotropy (processing). To this end, we simulated 1000 variants, in systems involving three, five, or ten traits, with a sample size of 10,000, 50,000, or 300,000, and a proportion of missing of either 0.0 or 0.3 (we introduce this factor because the presence of missing values has an impact on computational complexity). The computation time was measured 1000 times on a Dual-core Intel Core i5 processor with 2.3 GHz and 16 GB of RAM LPDDR3.

### Mapping loci with pleiotropic effect on metabolic-syndrome-related traits

Finally, we used pleiotest to perform a multi-trait GWA analysis to map loci with pleiotropic effects on seven continuous traits related to MetS: body mass index, systolic blood pressure, serum urate, glucose level, low-density lipoproteins, triglycerides, and creatinine. The traits serum urate and triglycerides were log-transformed to make their distribution symmetric. These traits were selected based on the articles used for comparison and data availability. Genotypes and phenotypes derived from distantly related white Europeans from the UK Biobank [18]. All phenotypes were centered and linearly adjusted by age, sex, smoking status, assessment center, and 10 SNP-derived PCs using single-trait least square regressions.

SNP-genotypes were from the UKBiobank Axiom Array [19] (820,967 SNPs); these SNPs were filtered by removing those with minor-allele frequency smaller than 0.1% and using a call rate of 95%. After filtering we retained 607,490 SNPs in autosomal chromosomes. Genotyping data were available on 316,411 distantly related white European participants. To identify these individuals, we first confirmed self-reported ethnicity with SNP-derived PCs, and then computed genomic-relationships $\left(G_{ij} \right.= p^{ - 1} \mathop{\sum}\nolimits_{k = 1}^p (x_{ik} - 2\theta _k)(x_{jk} - 2\theta _k)/2\theta _k(1 - \theta _k)$, where $\theta _k$ is the allele frequency at the *k*^th^ SNP) among white Europeans and retained only individuals that had genomic relationship smaller than 0.05. Genomic relationships were computed using the BGData R-package. For further details about the sample selection process refer to Kim et al [20]. Among the individuals included in the study, 46% were male and age ranged from 39 to 73, with an average (and SD) of 56.9 (7.99) years.

For each SNP we obtained *p* values for various degrees of pleiotropy; we considered an association to be significant if the *p* value was smaller than 1e^−8^, a standard genome-wide significance threshold that accounts for multiple tests across SNPs. Further adjustment for multiple testing within loci is not needed because the sequential nature of the test controls for it.

We report our results in terms of independent regions of significance. We obtained these regions using the *pleio_ideogram* function, included in the pleiotest package. This function uses *p* values and a genetic map to group significant results that are at a distance smaller than a user-specified threshold (we use 1 Mbp) into non-overlapping genomic regions, each harboring at least one significant association.

Finally, we used data on tissue-specific eQTL from GTEx [21] to test whether the variants detected in our study were enriched for cis-eQTLs (i.e., SNPs within ±1 Mbp of the transcriptional start site of each gene) on 48 human tissues for which GTEx offer data. We tested for significant enrichment using a hypergeometric test by tissue.

## Results

### Power and Type-I error rate

The simulation results (Table 1), which were based on 500 million MC-replicates, showed that both sLRT and pleiotest had highly accurate type I error rate control. There were no statistical differences between these tests in type I error rate in any of the scenarios tested. With a sample size of 3000 pleiotest was, on average, slightly more conservative than sLRT (i.e., smaller error rate, which translates to slightly larger -log10(error rate)); this was expected because pleiotest uses ***S*** instead of ${\hat{\boldsymbol{R}}}$. Increasing sample size to 10,000 resulted in virtually the same type I error rate on both methods (Table S1 in Supplementary materials). Finally, both methods were slightly more conservative in cases of low error correlations (0.2), but the empirical type-I error rates were in all cases close to the desired significance level.

**Table 1:** Type I error rate (in -log10 scale) of sLRT and pleiotest by effects-scenario (Ha_1_ or Ha_2_), error correlation (Cor), and significance level (α).

	Cor = 0.2				Cor = 0.8			
	Ha_1_		Ha_2_		Ha_1_		Ha_2_	
-log10(α)	sLRT	pleioR	sLRT	pleioR	sLRT	pleioR	sLRT	pleioR
8	7.65	7.73	8.23	8.40	8.02	8.11	8.24	8.24
	[7.41,7.94]	[7.46,8.05]	[7.76,8.91]	[7.85,9.32]	[7.65,8.50]	[7.70,8.68]	[7.78,8.93]	[7.78,8.93]
7	6.95	7.00	7.19	7.24	6.94	7.03	7.06	7.10
	[6.84,7.07]	[6.88,7.12]	[7.04,7.35]	[7.08,7.42]	[6.83,7.06]	[6.91,7.17]	[6.93,7.19]	[6.96,7.24]
6	5.96	6.02	6.08	6.14	5.95	6.03	5.99	6.04
	[5.92,5.99]	[5.98,6.06]	[6.04,6.13]	[6.10,6.19]	[5.91,5.99]	[5.99,6.07]	[5.95,6.03]	[6.00,6.08]
5	4.98	5.02	5.05	5.09	4.97	5.02	4.97	5.01
	[4.96,4.99]	[5.01,5.03]	[5.04,5.06]	[5.08,5.10]	[4.96,4.98]	[5.00,5.03]	[4.96,4.99]	[5.00,5.02]
4	3.98	4.01	4.02	4.05	3.98	4.01	3.99	4.01
	[3.98,3.98]	[4.01,4.02]	[4.02,4.03]	[4.05,4.05]	[3.98,3.99]	[4.01,4.02]	[3.98,3.99]	[4.01,4.01]
3	2.99	3.01	3.00	3.02	2.99	3.01	2.99	3.01
	[2.99,2.99]	[3.01,3.01]	[3.00,3.00]	[3.02,3.02]	[2.99,2.99]	[3.01,3.01]	[2.99,2.99]	[3.00,3.01]
2	1.99	2.00	2.00	2.00	1.99	2.00	2.00	2.00
	[1.99,1.99]	[2.00,2.00]	[2.00,2.00]	[2.00,2.00]	[1.99,1.99]	[2.00,2.00]	[2.00,2.00]	[2.00,2.00]

Results are based on 500 million Monte Carlo (MC) simulations with sample size 3000; 95% confident intervals between square brackets.

**Fig. 1:**
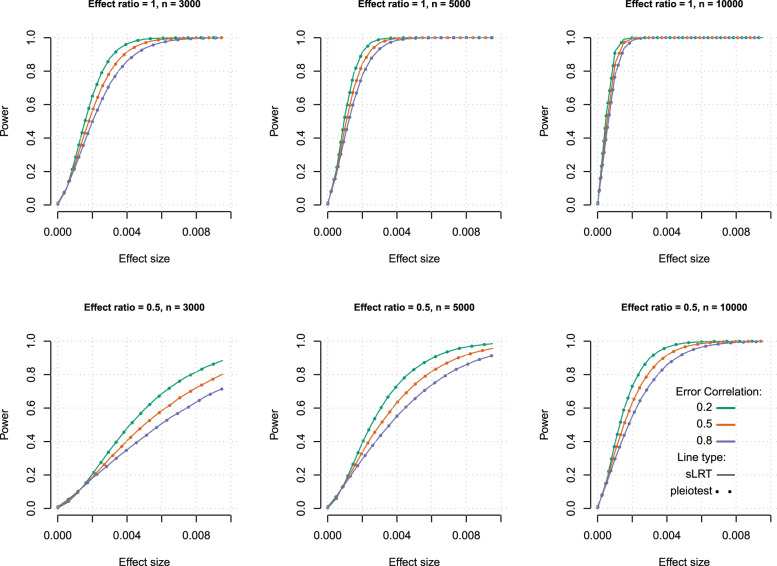
Power to detect pleiotropy of sLRT (thin solid line) and pleiotest (thick dashed line). Each plot corresponds to a different sample size and effect-size ratio (effect ratio = 1: $\beta _1 = \beta _2 = \beta _3 \,\ne\, 0$; effect ratio = 0.5: $0.5\beta _1 = \beta _2 = \beta _3 \,\ne\, 0$).

The results of the power analysis showed that sLRT and pleiotest had indistinguishable power across scenarios (Fig. 1). MC error is not displayed in Fig. 1 because with the number of replicates conducted, the MC-error was always smaller than 1% of the reported power. As it is expected, power increased effect- and sample-size. Holding everything else constant, the power was higher with low error correlation and effect ratio = 1 (i.e., the same effect size on both traits). In a suite of three traits, achieving high power to detect pleiotropy for an SNP with moderately small effect size (<0.2% of the phenotypic variance) required a sample size larger than 10,000 (The estimated MC error is in all scenarios < 0.0016). A large sample size such as the one of the UKBiobank (>300 K) or more produces high power even with small effect sizes (<0.2%).

### Computational performance

**Fig. 2:**
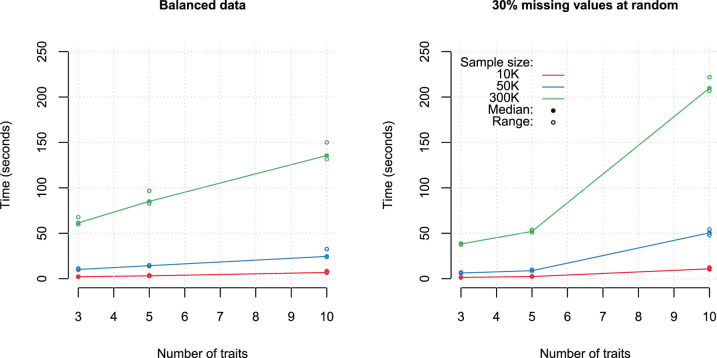
Total computational time (in seconds) to process 1000 variants with balanced and unbalanced data, and an increasing number of traits. Colors indicate sample size from 10,000 to 300,000.

For balanced data, the benchmark (Fig. 2) showed that the computational time required to perform the test scales approximately linearly with the number of traits and with sample size (left panel of Fig. 2); but these two factors interact, making the difference in the time required to process 5 or 10 traits larger for large sample size. When 30% of the data were missing at random, the computational time was no longer linear on the number of traits (right panel of Fig. 2); this happens because with completely random missingness the number of missing-value patterns present in the data grows exponentially with the number of traits (i.e., increasing data fragmentation). However, with real data, missing data often does not happen completely at random (e.g., some subjects lack data for a subset of the traits); in such cases the increase of computational time with missing records is expected to be smaller.

For balanced data and a biobank-sized sample (*n* = 300,000), it took on average ~85 seconds for pleiotest to analyze 1000 SNP for 5 traits. For systems involving 10 traits, the computational times required to process 1000 SNPs were about 50–60% higher than for analysis with the same sample size and 5 traits. This represents a remarkable computational improvement relative to the sLRT of the *pleio* R-package [22] that, with no missing values and *n* = 300,000, yielded a median computation time of 20.1, 25.8, and 44.6 s per variant for 3, 5, and 10 traits, respectively.

The benchmark presented in Fig. 2 shows that pleiotest can process an entire chromosome (~50,000 SNPs) with a very large sample size and five traits in about one hour and ten minutes (chromosomes can be process in parallel as separate jobs). For imputed genotypes, after QC and filtering, long chromosomes may include ~1million SNPs. Thus, for a sample size of 300,000 and 5 traits, processing a long chromosome in a single job would take about 23.5 h; but this task can also be parallelized (using features offered by the BEDMatrix package which is compatible with pleiotest) in, for example, jobs of 100,000 SNPs each, which will take <2.5 h to finish.

### Pleiotropic analysis of seven metabolic-syndrome-related phenotypes

Metabolic syndrome (MetS) is characterized by a group of conditions that are often co-morbid and are considered risk factors for cardiovascular disease (CVD) and type-2 diabetes [23]. MetS scoring systems are based on different measures of conditions such as obesity, vascular dysfunction, and inflammation, elevated plasma glucose, pro-thrombotic state, and/or atherogenic dyslipidemia [7]. We used pleiotest, with data from the UK-Biobank, for the multivariate GWA analysis of seven traits associated with MetS: body mass index (1; BMI), systolic blood pressure (2; SBP), serum urate (3; URA), glucose level (4; GLU), low-density lipoproteins (5; LDL), triglycerides (6; TRI), and creatinine (7; CRE). The correlations between the adjusted phenotypes ranged from −0.09 to 0.38 (Fig. S1 in supplemental material). Traits TRI, BMI, and URA clustered with phenotypic correlations above 0.27; the pairs of traits TRI - LDL, and URA - CRE had moderate correlations (0.30 and 0.25, respectively).

Our analyses of seven MetS-related traits found widespread pleiotropy, including 170 non-overlapping genomic regions (2982 SNPs) with pleiotropic effects in at least two traits (Table 2). TRI was the trait with the largest number of SNPs with simultaneous significant associations with it and at least another trait (1953); however, URA was the trait most often involved in regions exhibiting pleiotropic effects (99 segments involving SNPs with pleiotropic effects harbored SNPs with significant effects for URA). Overall, traits URA and CRE were involved in the largest number of regions (45) harboring SNPs significantly associated with both traits.

**Table 2:** Number of non-overlapping genomic regions (# of SNPs) with a significant effect on at least two traits.

	BMI	SBP	URA	GLU	LDL	TRI	CRE	Total
BMI		16 (44)	22 (268)	7 (96)	17 (83)	30 (334)	14 (56)	80 (881)
SBP	16 (44)		11 (25)	2 (4)	5 (22)	9 (121)	3 (18)	33 (234)
URA	22 (268)	11 (25)		4 (43)	23 (170)	27 (602)	45 (155)	99 (1263)
GLU	7 (96)	2 (4)	4 (43)		6 (17)	9 (165)	2 (7)	21 (332)
LDL	17 (83)	5 (22)	23 (170)	6 (17)		32 (648)	6 (25)	63 (965)
TRI	30 (334)	9 (121)	27 (602)	9 (165)	32 (648)		14 (83)	88 (1953)
CRE	14 (56)	3 (18)	45 (155)	2 (7)	6 (25)	14 (83)		70 (344)

**Fig. 3:**
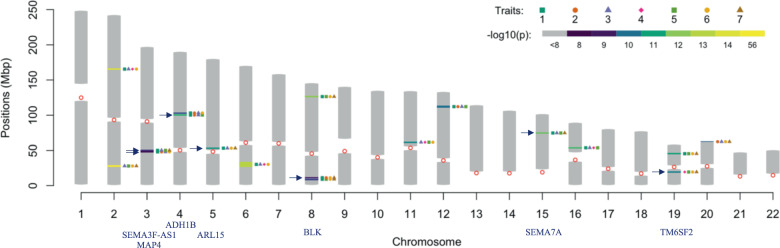
Ideogram of regions harboring SNPs with effects in at least four traits. The symbols by the region indicate the trait. Traits included in the analyses were: body mass index (1), systolic blood pressure (2), serum urate (3), glucose level (4), low-density lipoproteins (5), triglycerides (6), and creatinine (7). Blue arrows denote novel associations.

We also tested for higher-order pleiotropy, including three- and four-traits pleiotropy. We found 18 well-defined regions harboring (246) SNPs with significant associations with at least four traits (Fig. 3; Manhattan plots are in Fig. S3 of the supplemental material) but did not find any SNP with significant association with more than four traits. Table 3 shows the traits involved in four-traits pleiotropy by region, as well as the annotated genes corresponding to the SNPs with the lowest *p* values. BMI was the trait most often involved in regions with pleiotropic effects in at least four traits. Traits URA, TRI, and CRE were also highly represented in regions harboring SNPs with four-traits pleiotropy. The first region in the chromosome (CHR) 2, positioned between 26.8 and 28.6 Mbp, contains the SNPs with the lowest *p* values and harbors three important MetS-related genes: *GCKR*, *C2orf16*, and *ZNF512*. Many of the genes in Table 3 were already reported in associations with up to four metabolic traits, [24] but there are also seven regions that, according to the NHGRI-EBI catalog of human GWA studies (GWAS catalog; https://www.ebi.ac.uk/gwas/), do not contain SNPs reported in association with MetS. Two of these regions, which harbor genes *ARL15* (CHR 5) and *TM6SF2* (CHR 19), were reported in MetS studies [25, 26] that are not part of the GWAS catalog database. Therefore, we found five regions harboring SNPs with at least four MetS traits that have not been reported before. The most important genes (corresponding to the SNPs with the lowest *p* values) in these regions are *MAP4*, *SEMA3F-AS1*, *ADH1B*, *BLK*, and *SEMA7A*.

**Table 3:** Regions harboring SNPs with four-trait pleiotropic significant effects (*p* value < 1e^−8^).

Chromosome	GWAS Catalog^b^	SNP w/smallest *p* value in -log10 scale	Genes^c^	Traits						
[position]^a^				BMI	SBP	URA	GLU	LDL	TRI	CRE
2	Yes	rs1260326 (56.312)	GCKR C2orf16			×		×	×	×
[26.8–28.6]			ZNF512							
2	Yes	rs1128249 (13.556)	COBLL1	×		×	×		×	
[164.2–165.2]										
3	No	rs62260779 (8.429)	MAP4	×	×	×			×	
[47.5–48.5]										
3	No	rs2624847 (8.631)	SEMA3F-AS1	×		×		×		×
[49.6–50.6]										
4	No	rs1229984 (10.626)	ADH1B	×	×	×		×		
[98.8–99.8]										
4	Yes	rs13107325 (9.390)	SLC39A8	×	×	×			×	
[101.8–102.8]										
5	No^d^	rs4865796 (10.417)	ARL15	×		×			×	×
[53.5–54.5]										
6	Yes	rs1264377 (13.211)	DDR1	×		×	×		×	
[27.3–33.2]			NOTCH4							
8	Yes	rs898137 (8.854)	LOC157273	×				×	×	×
[8.6–10]										
8	No	rs13280813 (8.611)	BLK	×	×				×	×
[11.1–12]										
8	Yes	rs2001945 (11.847)	TRIB1	×				×	×	×
[125–126]										
11	Yes	rs174547 (10.284)	FADS1			×	×	×	×	
[61.3–62.3]			FADS2							
12	Yes	rs653178 (9.673)	ATXN2	×	×	×		×		
[111–112.6]										
15	No	rs11856835 (10.799)	SEMA7A	×		×		×		×
[73.9–74.9]										
16	Yes	rs1421085 (10.767)	FTO	×		×	×	×		
[53.3–54.3]										
19	No^d^	rs58542926 (9.965)	TM6SF2				×	×	×	×
[18.8–19.8]										
19	Yes	rs4420638 (10.508)	APOC1	×				×	×	×
[44.4–45.4]			TOMM40							
20	Yes	rs8121509 (9.408)	OPRL1		×	×			×	×
[63.6–64.3]										

*BMI* body mass index, *SBP* systolic blood pressure, *URA* serum urate, *GLU* glucose level, *LDL* low-density lipoprotein, *TRI* triglycerides, *CRE* creatinine.

^a^Position in mega base-pairs (Mbp).

^b^Whether at least one SNP in the region has been reported for MetS in the GWAS catalog.

^c^Gene corresponding to the SNP with the smallest *p* value.

^d^Reported elsewhere.

To further determine which of the associations found in our study were previously reported, we compared our findings against the GWAS catalog and two specific studies, Avery et al. [7] and Kraja et al. [24]; the former used principal components derived from multiple MetS-related traits as phenotypes for the GWA, and the latter published a meta-analysis of eight metabolic traits and six inflammatory markers related to MetS. Table 4 shows the number of SNPs and genomic regions that matched our findings and these studies. Fifty-six percent of the SNPs we found to have pleiotropic effects in at least two traits were located at a distance smaller than 1-Mbp to SNPs reported for MetS in the GWAS catalog. Only 30% of the 170 regions with two-traits pleiotropy included SNPs reported in the GWAS catalog, and only 8% were reported by Avery et al. or by Kraja et al. Of the 18 pleiotropic regions reported in Table 3, only 6 and 8 contained SNPs reported by Avery et al. and Kraja et al., respectively. Differences in sample sizes, in the methods used for association analyses (e.g., Avery et al. use a PC-based method that can be underpowered to find associations involving only a few traits of the system), as well as the thresholds used to determine significance (e.g., Avery et al. used a higher *p* value threshold of 2.13e^−7^ than the one used here, namely 1e^−^^8^) could explain the partial overlap of findings.

**Table 4:** Number and percentage of discoveries in our study (*p* value < 1e^−8^) that have been previously reported, by number of traits simultaneously affected (variants with pleiotropic effects in at least 2, 3, or 4 traits).

Number of traits simultaneously affected	Number of discoveries in this study	Overlap with other studies^a^		
		GWAS catalog	Avery et al. (2011)	Kraja et al. (2014)
Non-overlapping genomic regions				
2	170	51 (30%)	14 (8%)	13 (8%)
3	44	22 (50%)	6 (14%)	8 (18%)
4	18	11 (61%)	6 (33%)	8 (44%)
SNPs				
2	2982	1677 (56%)	305 (10%)	1081 (36%)
3	871	497 (57%)	95 (11%)	329 (38%)
4	246	202 (82%)	54 (22%)	130 (53%)

^a^SNPs reported in other studies that were within a 1-Mbp of a discovery in our study were considered overlapping.

Finally, using data from the gene expression data (GTEx) portal, [21] we found that the SNPs with pleiotropic effects on at least four traits reported in this study were enriched for eQTL in 41 of the 48 tissues for which GTEx eQTL results are available (Table S3 and Fig. S3 of the Supplementary materials). We also found that the genes listed in Table 3 had distinctive average expression patterns across tissues (Fig. S4). Not surprisingly, the genes located in regions which we found to be involved in four-traits pleiotropy tended to be over-expressed in the liver.

## Discussion

Quantitative genetic studies [2] and GWA analyses [3] suggest that pleiotropy is a widespread phenomenon. Pleiotropy drives genetic correlations between traits and underlies the concurrent development of multiple diseases. Therefore, identifying risk loci with a pleiotropic effect is an important step towards understanding the genetic basis of many syndromes. However, genome-wide systematic mapping of loci with pleiotropic effects is both statistically and computationally challenging. Conceptually, one could identify loci with pleiotropic effects by identifying SNPs that have significant associations with more than one trait using *p* values from single-trait association analysis. However, defining significance with this approach is challenging because it is not clear how to determine the number of independent tests being performed. This happens because the tests statistics are often correlated between traits; as a result, accurate error control is challenging. To address this, Schaid et al. [9] proposed a formal (sequential) test for pleiotropy that provides both adequate error control and the power achieved with multi-trait models. Simulations provided by Schaid et al. and in the present study confirm that the sequential test is powerful and offers adequate error control. Additionally, the sequential test groups a potentially large number of pleiotropic configurations (i.e., many alternative hypotheses) into a smaller number of meaningful pleiotropic states defined by the number of traits involved (e.g., pleiotropy involving at least *q* traits for *q* = 2, …, *p*); this facilitates the communication and interpretation of results. However, the computation of the required test statistics as proposed by Schaid et al., does not scale well to large sample size and hundreds of thousands (possibly millions) of SNPs. Therefore, to overcome those limitations we developed an approximation to the test and software package (*pleiotest*) that scales to big data. Our approach uses a Wald’s test instead of a likelihood ratio test and, more importantly, an approximation to the residual (co)variance matrix, which reduces the computational burden of the test and provides some computational improvements.

The simulations presented in this study show that the proposed approximation (pleiotest) has adequate error control and achieves the same power of the original sequential test. In systems involving many traits and with a small sample size (e.g., *n* < 3000) our approach could be slightly conservative; however, this does not deteriorate power because the test is only conservative in situations in which power is very high (e.g., when a SNP explains 1% of the variance or more).

Conducting large-scale GWA studies requires efficient computational strategies. We achieve this by two means. Firstly, we implemented the proposed test of pleiotropy using the C++ language and several computational strategies aiming at optimizing the software’s performance. Secondly, we leveraged software packages previously developed by our group (the BEDMatrix package [14]) which implements memory mapping of PLINK’s .bed files [27], thus offering the possibility of performing analyses on extremely large datasets using commodity software within the R-environment and without having to subset genotype files. Together, these strategies make our software suitable for analysis of systems involving many traits (e.g., up to 10 or 15, depending on the tolerable computation time) with very large sample size.

Unlike existing software [28] for Seemingly Unrelated Regressions (SUR), our package can be used with unbalanced data (i.e., cases in which some individuals may not have data for some traits). Our approach computes the likelihood of the observed data, under the assumption of non-informative missingness (i.e., the probability that a record is missing does not depend on the trait value, covariates, or the values of other traits). Computing the likelihood of unbalanced data can be challenging. To address this challenge our strategy is to group observations according to their missing-value pattern, generate the required summary statistics for each group, and then combine these statistics to derived estimates, SEs and *p* values. This approach is computationally efficient when the number of missing-value groups is small (e.g., when a sizable number of observations share the same missing-value pattern). However, the approach can involve a substantial computational burden when there are many missing-value groups, each involving a small number of observations. To avoid substantial increases in computation time due to highly fragmented data, pleiotest offers the option of dropping groups involving a small number of observations (e.g., *n* < 50).

The package also offers the possibility to set an early stop to the sequential tests if *p* values are higher than a user-specified threshold (e.g., 0.01), which also saves computation time. Finally, the algorithm offers the possibility of processing variants in parallel at multiple cores within a single R session. However, for a large-scale analysis conducted in high-performance computing clusters it may be more effective to parallelize the analysis into multiple jobs, each processing a chunk of DNA (e.g., tens of thousands of variants) in a single core – our software also offers this possibility.

The test implemented in this work assumes that traits follow a multivariate normal distribution. For analysis of quantitative traits this is not a major limitation because traits with strong departure from normality can be transformed and because with a large sample size, the Central Limit Theorem guarantees the normality of estimates obtained from linear models [29]. Schaid et al. [11] presented an extension of the sequential testing to generalized linear models. Further work is needed to adapt the approach presented in this study to such a class of models. In the meantime, a possible approximation is to first use single-trait generalized linear models to “adjust” traits by (co)variates and then use residuals (e.g., z-scores or deviance residuals) as traits in our software.

Deng and Pan [30] presented an implementation of the sequential test for pleiotropy that uses summary statistics. The authors considered both standard pleiotropy as well as analyses conditional on other traits. Tests based on summary statistics are appealing; implementing them requires approximating the error (co)variance matrix (which in the case of Deng and Pan is done based on Z-statistics) and assuming Hardy-Weinberg equilibrium. When the data for each of the traits originate either from the same set of subjects (complete data) or from completely different cohorts (no overlap in samples) the implementation of the test is straightforward. However, implementing the test based on summary statistics becomes challenging when the data used to generate the summary statistics originate from partially overlapping samples (i.e., when there is multiple “missing value patterns”). In this case, the error (co)variance matrix depends on the sample size of each of the missing value patterns. Unfortunately, this information is often not available–these difficulties and possible approximations are discussed in Deng and Pan [30].

We used pleiotest to map variants with pleiotropic effects on seven MetS-related traits. We found abundant evidence of pleiotropy: 170, 44, and 18 independent genomic regions that contained significant associations with at least two, three, and four of the seven traits studied, respectively. These regions cover many previously reported findings as well as many novel ones. As expected, the proportion of shared risk loci between any pair of traits was directly related to the phenotypic correlation between traits (see Figs. S1 and S2); however, two pairs, LDL-TRI and URA-TRI, stand-out as cases with a proportion of pleiotropic loci that is much higher than what one would predict based on the linear trend relating the proportion of pleiotropic loci and the phenotypic correlation.

The SNPs discovered to have at least 4-trait pleiotropy (see Table 3) are located in regions that include important genes; for example, the smallest-*p* value in our study corresponds to a SNP in *GCKR* which has been previously linked to MetS by Avery et al. [7], as well as by Kraja et al. [24]. Genes *C2orf16*, *ZNF512* and *COBLL1* were also previously reported [24, 31, 32], as was *SLC39A8* in chromosome (CHR) 4, which plays a role in hypertension and has been found to be associated to type-2 diabetes [33]. Genes *ARL15* (CHR 5) and *TM6SF2* (CHR 19) were not included in the GWAS catalog as MetS-associated genes; however, these genes were reported elsewhere [25, 26]; thus, we do not consider these genes novel discoveries of our study in relation to MetS.

Chromosome 6 harbors the longest genomic region (~6-Mbp) containing SNPs with pleiotropic effects on MetS-traits. This region involves many BMI-related genes, such as *DDR1* and *NOTCH4*. Genes *FADS1* and *FADS2* in CHR 11 encode fatty acid desaturase enzymes and were reported affecting LDL cholesterol levels and MetS [34]. Other genes reported for MetS are *ATXN2* (CHR 12), *FTO* (CHR 16), *APOC1*, and *TOMM40* (CHR 19) [35].

In addition to confirming previous findings, our analysis of MetS-related traits identified *five novel regions* associated with at least four MetS-related traits. These regions harbor multiple genes potentially associated with MetS including genes *MAP4* and *SEMA3F-AS1* (both in CHR 3), *ADH1B* (CHR 4), *BLK* (CHR 8), and *SEMA7A* (CHR 15). While these genes have not been directly related to MetS, some have been implicated with other MetS-related traits and behaviors such as SBP (gene *MAP4* [36]), alcohol dependence, and gout (gene *ADH1B* [37]), rheumatoid arthritis, systemic sclerosis, and the immune response to smallpox vaccine (gene *BLK*), and neuronal processes and the risk of acute atherothrombotic stroke (gene *SEMA7A* [38]).

Gianola et al. [39] noted that linkage disequilibrium (LD) could lead to spurious findings about pleiotropy. Specifically, the authors noted that it is theoretically possible for two casual variants with effects on separate traits (i.e., without pleiotropic effects) to be in mutual LD with one or more SNPs. In those cases, there is no pleiotropy, but an association analysis may falsely identify some loci as being associated with more than one trait. This general problem also applies to the methods presented in this study. However, the level of LD among variants in the genotype array used in our study drops rapidly with physical distance, thus, reducing the likelihood of such apparent pleiotropy to be a major driver of the findings reported here.

## Supplementary information

Supplemental material
