# Supplementary material for: Mapping pleiotropic loci using a fast-sequential testing algorithm
Source: Eur J Hum Genet. 2021 Jun 18;29(12):1762–73. doi: 10.1038/s41431-021-00911-z (PMC8633382; doi:10.1038/s41431-021-00911-z)
Supplement: Supplementary file 1 — Supplemental material [file 41431_2021_911_MOESM1_ESM.docx]

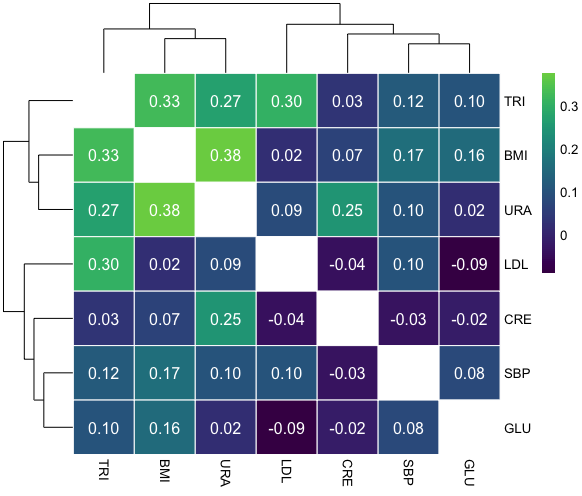


**Figure S1.** Correlations between traits: body mass index (BMI), systolic blood pressure (SBP), log-serum urate (URA), glucose level (GLU), log-density lipoproteins (LDL), log-triglycerides (TRI), and creatinine (CRE).

**Figure S2.** Proportion of shared risk loci (i.e., # SNPs associated with both traits / Number of SNPs associated with at least one of the two traits) versus sample correlation of the adjusted phenotypes.


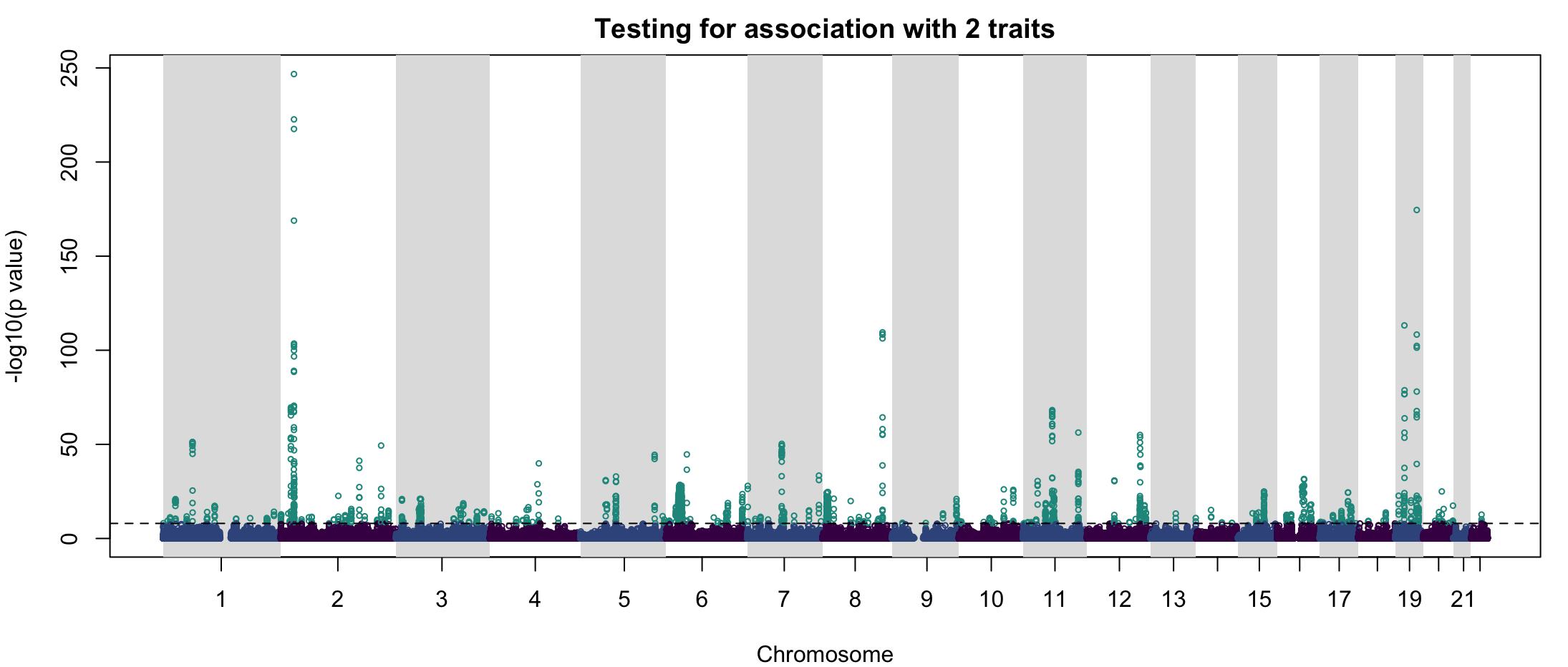


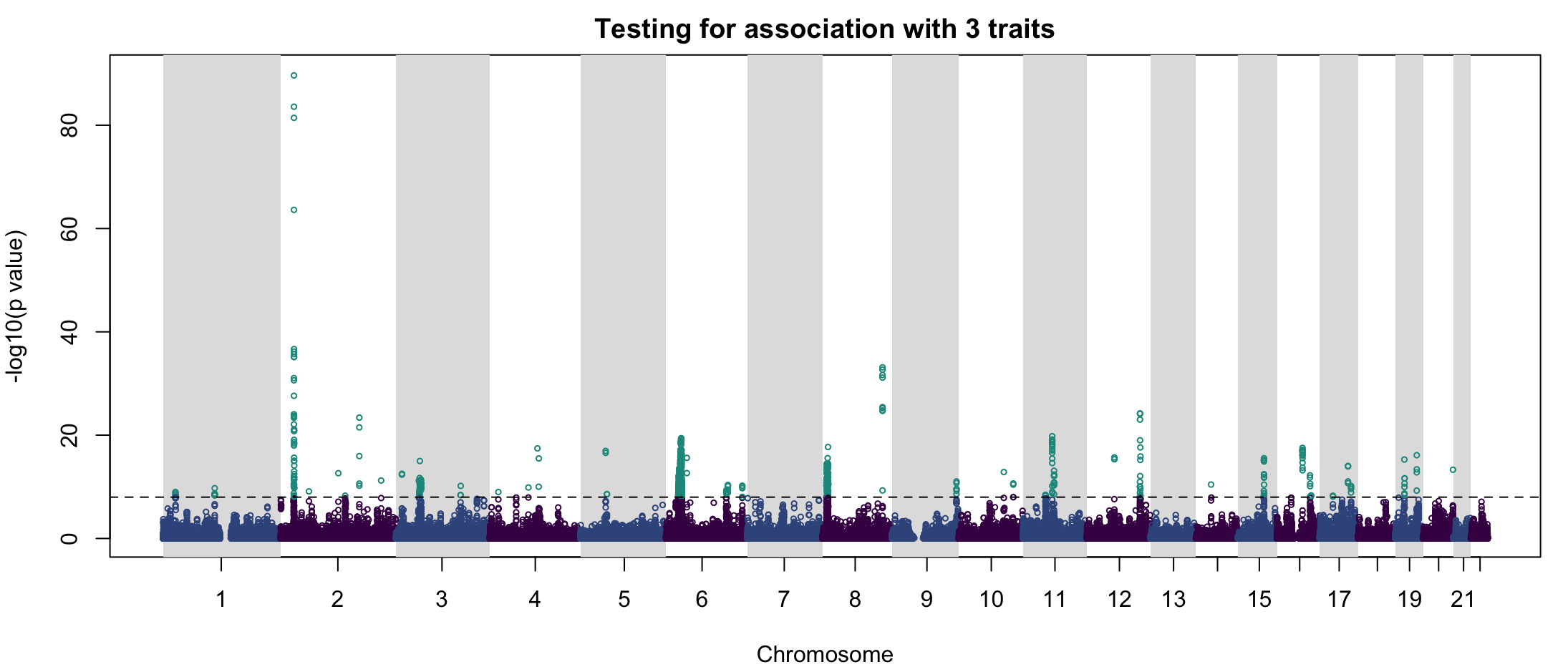

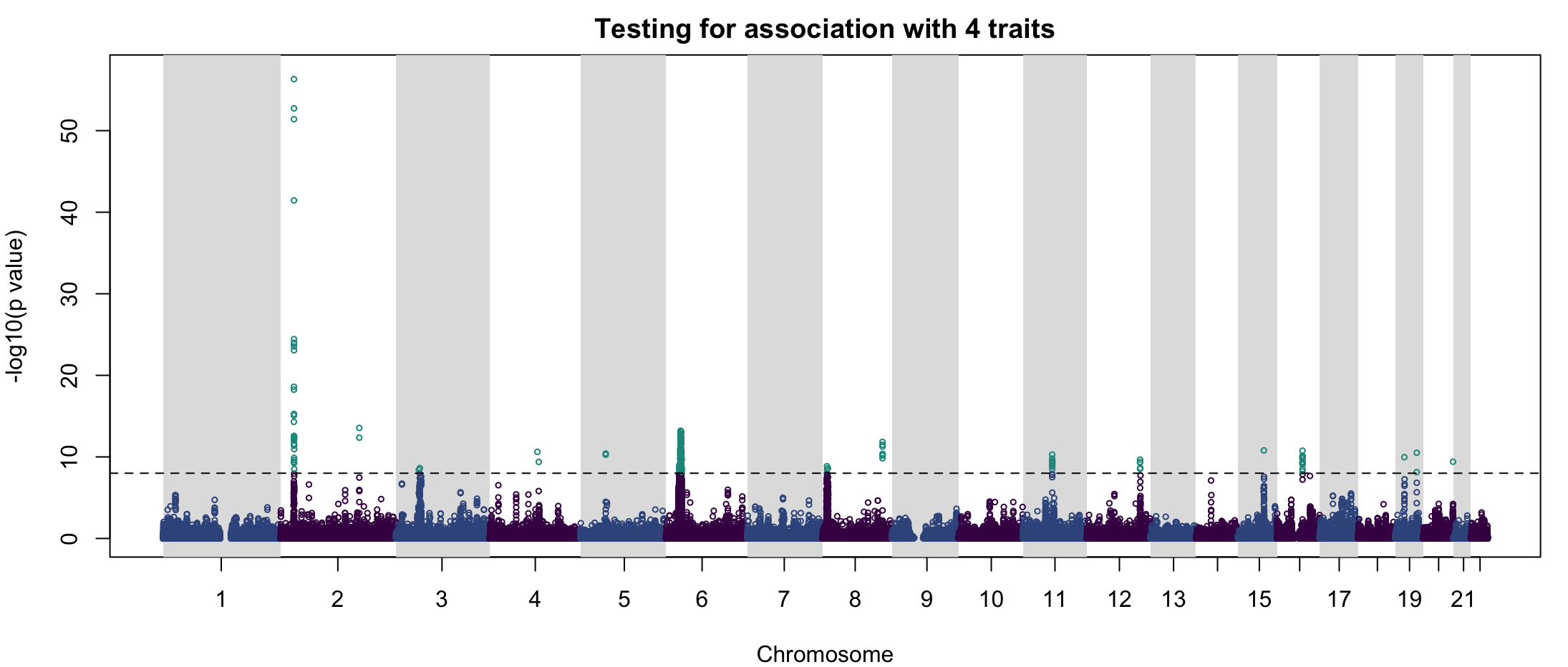


**Figure S3.** Manhattan plots of p-values testing for associations with two to four traits. The threshold for genome-wide significance is 1×10^-8^.

**Table S1.** Type I error rate (in -log10 scale) of sLRT and pleiotest by effects-scenario (Ha_1_ or Ha_2_), error correlation (Cor), and significance level (α). Results are based on 100 million Monte Carlo (MC) simulations with sample size 10,000; 95% confident intervals between square brackets.

|  | Cor = 0.2 | | | | | Cor = 0.8 | | | | |
| --- | --- | --- | --- | --- | --- | --- | --- | --- | --- | --- |
|  | Ha_1_ | | Ha_2_ | | Ha_1_ | | | Ha_2_ | |  |
| -log10(α) | sLRT | pleiotest | sLRT | pleiotest | sLRT | | pleiotest | sLRT | pleiotest |  |
| 8 | NA* | NA | NA | NA | 8.08 | | 8.08 | 7.31 | 7.31 |  |
|  | NA | NA | NA | NA | [7.34,9.68] | | [7.34,9.68] | [6.56,8.90] | [6.56,8.90] |  |
| 7 | 6.93 | 6.93 | 7.18 | 7.18 | 6.8 | | 6.8 | 6.83 | 6.83 |  |
|  | [6.67,7.25] | [6.67,7.25] | [6.81,7.66] | [6.81,7.66] | [6.61,7.02] | | [6.61,7.02] | [6.37,7.52] | [6.37,7.52] |  |
| 6 | 5.98 | 6.00 | 5.99 | 6.01 | 5.93 | | 5.94 | 5.97 | 5.99 |  |
|  | [5.89,6.08] | [5.91,6.10] | [5.89,6.09] | [5.91,6.11] | [5.86,6.00] | | [5.86,6.01] | [5.79,6.17] | [5.80,6.19] |  |
| 5 | 5.00 | 5.01 | 4.99 | 5.01 | 4.98 | | 4.99 | 4.99 | 5.01 |  |
|  | [4.97,5.03] | [4.98,5.04] | [4.96,5.02] | [4.97,5.04] | [4.95,5.00] | | [4.97,5.01] | [4.93,5.06] | [4.95,5.07] |  |
| 4 | 4.00 | 4.00 | 3.99 | 4.00 | 3.99 | | 4.00 | 3.99 | 3.99 |  |
|  | [3.99,4.00] | [3.99,4.01] | [3.98,4.00] | [3.99,4.01] | [3.98,4.00] | | [3.99,4.00] | [3.97,4.01] | [3.97,4.01] |  |
| 3 | 2.99 | 3.00 | 3.00 | 3.00 | 2.99 | | 3.00 | 2.99 | 3.00 |  |
|  | [2.99,3.00] | [3.00,3.00] | [2.99,3.00] | [3.00,3.01] | [2.99,3.00] | | [3.00,3.00] | [2.99,3.00] | [2.99,3.00] |  |
| 2 | 2.00 | 2.00 | 2.00 | 2.00 | 2.00 | | 2.00 | 2.00 | 2.00 |  |
|  | [2.00,2.00] | [2.00,2.00] | [2.00,2.00] | [2.00,2.00] | [2.00,2.00] | | [2.00,2.00] | [2.00,2.00] | [2.00,2.00] |  |

* Empty cells correspond to settings for which there were less than 2 rejections.

**Table S2.** Computation time (in seconds) used in data pre-processing, solving equations, and performing the sequential test for pleiotropy (Processing) using the *pleiotest* package. Median and time range are presented to analyze 1,000 variants from 3, 5 or 10 traits; 10K, 50K or 300K sample size, and proportion of missing values (i.e., Prop. NA) of 0 or 0.3. Each simulation was replicated 100 times.

| # of Traits | Sample Size | Prop. NA | Pre-processing | Processing | Total |
| --- | --- | --- | --- | --- | --- |
| 3 | 10K | 0 | 0.11 [0.10 - 0.13] | 2.06 [1.98 - 2.20] | 2.17 [2.10 - 2.33] |
| 3 | 10K | 0.3 | 0.08 [0.07 - 0.28] | 1.29 [1.21 - 1.45] | 1.43 [1.29 - 1.55] |
| 3 | 50K | 0 | 0.61 [0.57 - 0.79] | 9.52 [9.18 - 10.39] | 10.16 [9.79 - 11.17] |
| 3 | 50K | 0.3 | 0.45 [0.40 - 0.52] | 5.83 [5.57 - 6.42] | 6.27 [6.00 - 6.89] |
| 3 | 300K | 0 | 4.93 [4.68 - 5.57] | 56.48 [54.93 - 62.30] | 61.45 [59.72 - 67.68] |
| 3 | 300K | 0.3 | 3.57 [3.39 - 3.90] | 34.71 [33.88 - 35.51] | 38.28 [37.38 - 39.00] |
| 5 | 10K | 0 | 0.13 [0.12 - 0.20] | 2.99 [2.90 - 3.44] | 3.14 [3.04 - 3.63] |
| 5 | 10K | 0.3 | 0.14 [0.13 - 0.40] | 2.21 [2.09 - 2.41] | 2.36 [2.23 - 2.60] |
| 5 | 50K | 0 | 0.76 [0.66 - 0.98] | 13.53 [13.09 - 14.02] | 14.32 [13.78 - 14.85] |
| 5 | 50K | 0.3 | 0.88 [0.78 - 1.18] | 7.89 [7.68 - 9.04] | 8.80 [8.48 - 9.91] |
| 5 | 300K | 0 | 5.63 [5.33 - 7.20] | 79.53 [77.30 - 89.58] | 85.15 [82.74 - 96.78] |
| 5 | 300K | 0.3 | 6.11 [5.83 - 6.38] | 45.88 [45.08 - 47.72] | 52.03 [50.92 - 53.80] |
| 10 | 10K | 0 | 0.18 [0.17 - 0.25] | 6.64 [6.42 - 8.12] | 6.84 [6.62 - 8.37] |
| 10 | 10K | 0.3 | 0.75 [0.70 - 0.89] | 10.09 [9.52 - 11.45] | 10.84 [10.26 - 12.27] |
| 10 | 50K | 0 | 1.04 [0.95 - 3.65] | 23.42 [22.95 - 28.97] | 24.51 [23.97 - 32.62] |
| 10 | 50K | 0.3 | 9.55 [9.27 - 10.90] | 40.86 [38.45 - 44.46] | 50.48 [47.99 - 54.27] |
| 10 | 300K | 0 | 7.47 [7.06 - 18.43] | 128.08 [124.17 - 132.32] | 135.62 [131.45 - 150.08] |
| 10 | 300K | 0.3 | 91.42 [89.81 - 101.34] | 118.00 [115.54 - 122.78] | 209.84 [206.56 - 221.86] |

**Table S3.** Enrichment p-value by tissue calculated with a hyper-geometric test using GTEx data.

| Tissue | #  findings^a^ | #  eQTLs^b^ | # sig. both^c^ | p-value |
| --- | --- | --- | --- | --- |
| Heart Atrial Appendage | 244 | 15610 | 166 | 1.62E-199 |
| Skin Not Sun Exposed Suprapubic | 244 | 24609 | 177 | 4.35E-186 |
| Muscle Skeletal | 244 | 26171 | 180 | 9.35E-186 |
| Lung | 244 | 22194 | 172 | 4.92E-185 |
| Thyroid | 244 | 32594 | 187 | 5.37E-182 |
| Artery Aorta | 244 | 19075 | 160 | 2.42E-174 |
| Colon Transverse | 244 | 14967 | 150 | 3.70E-173 |
| Adipose Subcutaneous | 244 | 27353 | 174 | 8.71E-173 |
| Skin Sun Exposed Lower leg | 244 | 29018 | 175 | 3.34E-170 |
| Artery Tibial | 244 | 28077 | 172 | 2.53E-167 |
| Adipose Visceral Omentum | 244 | 19692 | 155 | 1.01E-163 |
| Brain Cerebellum | 244 | 11680 | 134 | 5.39E-160 |
| Liver | 244 | 5455 | 112 | 2.53E-158 |
| Heart Left Ventricle | 244 | 13776 | 138 | 1.94E-156 |
| Nerve Tibial | 244 | 31072 | 169 | 1.56E-155 |
| Esophagus Muscularis | 244 | 23672 | 155 | 1.89E-151 |
| Esophagus Mucosa | 244 | 23979 | 154 | 1.57E-148 |
| Cells Cultured fibroblasts | 244 | 27003 | 150 | 2.80E-134 |
| Pituitary | 244 | 10152 | 114 | 1.36E-132 |
| Breast Mammary Tissue | 244 | 15659 | 121 | 7.66E-122 |
| Testis | 244 | 23565 | 133 | 2.84E-119 |
| Spleen | 244 | 10301 | 102 | 2.31E-111 |
| Pancreas | 244 | 12640 | 107 | 6.59E-110 |
| Adrenal Gland | 244 | 7983 | 87 | 8.67E-98 |
| Stomach | 244 | 10899 | 94 | 1.23E-96 |
| Esophagus Gastroesophageal Junction | 244 | 14665 | 101 | 8.07E-95 |
| Colon Sigmoid | 244 | 13458 | 93 | 7.21E-87 |
| Brain Cerebellar Hemisphere | 244 | 8475 | 78 | 8.34E-82 |
| Prostate | 244 | 7007 | 72 | 1.30E-78 |
| Cells EBV-transformed lymphocytes | 244 | 3588 | 52 | 9.70E-64 |
| Brain Caudate basal ganglia | 244 | 7048 | 58 | 1.00E-57 |
| Kidney Cortex | 244 | 428 | 27 | 2.91E-52 |
| Brain Frontal Cortex BA9 | 244 | 5718 | 50 | 3.93E-51 |
| Vagina | 244 | 2472 | 26 | 1.81E-29 |
| Brain Hypothalamus | 244 | 4344 | 29 | 5.39E-27 |
| Brain Amygdala | 244 | 2353 | 22 | 3.07E-24 |
| Brain Hippocampus | 244 | 3854 | 19 | 5.33E-16 |
| Brain Cortex | 244 | 8515 | 20 | 1.19E-10 |
| Small Intestine Terminal Ileum | 244 | 4641 | 13 | 1.54E-08 |
| Brain Anterior cingulate cortex BA24 | 244 | 3801 | 8 | 3.67E-05 |
| Artery Coronary | 244 | 6126 | 10 | 6.75E-05 |
| Ovary | 244 | 4420 | 5 | 1.14E-02 |
| Brain Putamen basal ganglia | 244 | 5268 | 5 | 2.39E-02 |
| Brain Nucleus accumbens basal ganglia | 244 | 7192 | 5 | 8.13E-02 |
| Minor Salivary Gland | 244 | 3188 | 1 | 3.86E-01 |
| Brain Substantia nigra | 244 | 1825 | 0 | 5.32E-01 |
| Uterus | 244 | 2245 | 0 | 6.12E-01 |
| Brain Spinal cord cervical c-1 | 244 | 2878 | 0 | 6.98E-01 |

^a^ Number of significant SNPs in our study that have data in GTEx; ^b^ Number of eQTLs; ^c^ Number of significant SNPs in our study that are eQTLs in GTEx.

**Table S4.** Trait-specific SNP estimates, standard errors (SE), and p-values derived from multi-trait GLS regression.

| BMI | | | |
| --- | --- | --- | --- |
| SNP ID | Estimate | SE | P-value |
| rs1260326 | -0.05594 | 0.01223 | 4.76E-06 |
| rs1128249 | 0.05780 | 0.01223 | 2.28E-06 |
| rs62260779 | 0.10493 | 0.02001 | 1.58E-07 |
| rs2624847 | 0.10515 | 0.01364 | 1.29E-14 |
| rs1229984 | -0.20893 | 0.04015 | 1.96E-07 |
| rs13107325 | 0.26479 | 0.02275 | 2.66E-31 |
| rs4865796 | 0.05682 | 0.01295 | 1.15E-05 |
| rs1264377 | 0.09196 | 0.01547 | 2.79E-09 |
| rs898137 | 0.06806 | 0.01197 | 1.31E-08 |
| rs13280813 | -0.07527 | 0.01195 | 2.99E-10 |
| rs2001945 | -0.06402 | 0.01196 | 8.57E-08 |
| rs174547 | 0.04674 | 0.01256 | 1.99E-04 |
| rs653178 | -0.05215 | 0.01195 | 1.27E-05 |
| rs11856835 | -0.05460 | 0.01192 | 4.63E-06 |
| rs1421085 | 0.35494 | 0.01218 | 1.64E-186 |
| rs58542926 | -0.03431 | 0.02256 | 1.28E-01 |
| rs4420638 | -0.08164 | 0.01521 | 8.06E-08 |
| rs8121509 | 0.04117 | 0.01200 | 6.04E-04 |
| SBP | | | |
| SNP ID | Estimate | SE | P-value |
| rs1260326 | 0.10442 | 0.04882 | 3.24E-02 |
| rs1128249 | -0.20389 | 0.04882 | 2.96E-05 |
| rs62260779 | 0.35930 | 0.07996 | 7.02E-06 |
| rs2624847 | 0.07433 | 0.05453 | 1.73E-01 |
| rs1229984 | -1.07298 | 0.15957 | 1.77E-11 |
| rs13107325 | -0.53522 | 0.09086 | 3.85E-09 |
| rs4865796 | -0.20558 | 0.05171 | 7.01E-05 |
| rs1264377 | -0.01463 | 0.06183 | 8.13E-01 |
| rs898137 | 0.21194 | 0.04780 | 9.27E-06 |
| rs13280813 | -0.35296 | 0.04770 | 1.37E-13 |
| rs2001945 | 0.09440 | 0.04774 | 4.80E-02 |
| rs174547 | -0.03503 | 0.05017 | 4.85E-01 |
| rs653178 | 0.36898 | 0.04771 | 1.04E-14 |
| rs11856835 | 0.21926 | 0.04760 | 4.10E-06 |
| rs1421085 | 0.23546 | 0.04863 | 1.29E-06 |
| rs58542926 | 0.18194 | 0.08997 | 4.31E-02 |
| rs4420638 | 0.11643 | 0.06078 | 5.54E-02 |
| rs8121509 | -0.27650 | 0.04793 | 7.98E-09 |
| URA | | | |
| SNP ID | Estimate | SE | P-value |
| rs1260326 | 0.01267 | 0.00059 | 4.99E-102 |
| rs1128249 | -0.00415 | 0.00059 | 1.93E-12 |
| rs62260779 | 0.00677 | 0.00097 | 2.58E-12 |
| rs2624847 | 0.00602 | 0.00066 | 6.19E-20 |
| rs1229984 | 0.01484 | 0.00194 | 2.05E-14 |
| rs13107325 | -0.00644 | 0.00110 | 4.64E-09 |
| rs4865796 | -0.00428 | 0.00063 | 7.48E-12 |
| rs1264377 | 0.00576 | 0.00075 | 1.26E-14 |
| rs898137 | 0.00066 | 0.00058 | 2.55E-01 |
| rs13280813 | 0.00026 | 0.00058 | 6.49E-01 |
| rs2001945 | 0.00151 | 0.00058 | 8.72E-03 |
| rs174547 | 0.00405 | 0.00061 | 2.59E-11 |
| rs653178 | 0.00770 | 0.00058 | 1.43E-40 |
| rs11856835 | 0.00399 | 0.00058 | 4.30E-12 |
| rs1421085 | 0.00400 | 0.00059 | 1.07E-11 |
| rs58542926 | 0.00458 | 0.00109 | 2.71E-05 |
| rs4420638 | -0.00394 | 0.00073 | 7.90E-08 |
| rs8121509 | -0.00288 | 0.00058 | 6.50E-07 |
| GLU | | | |
| SNP ID | Estimate | SE | P-value |
| rs1260326 | -0.03449 | 0.00328 | 6.72E-26 |
| rs1128249 | -0.01995 | 0.00327 | 1.11E-09 |
| rs62260779 | 0.01990 | 0.00537 | 2.09E-04 |
| rs2624847 | 0.01187 | 0.00366 | 1.17E-03 |
| rs1229984 | -0.01361 | 0.01075 | 2.06E-01 |
| rs13107325 | 0.00617 | 0.00610 | 3.12E-01 |
| rs4865796 | -0.00431 | 0.00347 | 2.15E-01 |
| rs1264377 | 0.02167 | 0.00415 | 1.74E-07 |
| rs898137 | -0.00419 | 0.00321 | 1.91E-01 |
| rs13280813 | -0.00147 | 0.00320 | 6.46E-01 |
| rs2001945 | 0.00032 | 0.00321 | 9.20E-01 |
| rs174547 | -0.01444 | 0.00337 | 1.78E-05 |
| rs653178 | 0.00000 | 0.00320 | 1.00E+00 |
| rs11856835 | 0.00113 | 0.00319 | 7.24E-01 |
| rs1421085 | 0.02266 | 0.00327 | 3.93E-12 |
| rs58542926 | 0.02962 | 0.00605 | 9.80E-07 |
| rs4420638 | -0.00999 | 0.00408 | 1.43E-02 |
| rs8121509 | -0.00869 | 0.00322 | 6.91E-03 |
| LDL | | | |
| SNP ID | Estimate | SE | P-value |
| rs1260326 | 0.03216 | 0.00228 | 5.53E-45 |
| rs1128249 | -0.01142 | 0.00228 | 5.75E-07 |
| rs62260779 | -0.00973 | 0.00374 | 9.32E-03 |
| rs2624847 | -0.01185 | 0.00255 | 3.32E-06 |
| rs1229984 | -0.05096 | 0.00751 | 1.15E-11 |
| rs13107325 | -0.01651 | 0.00425 | 1.03E-04 |
| rs4865796 | 0.00712 | 0.00242 | 3.26E-03 |
| rs1264377 | -0.01061 | 0.00289 | 2.40E-04 |
| rs898137 | 0.01242 | 0.00224 | 2.82E-08 |
| rs13280813 | -0.00987 | 0.00223 | 9.74E-06 |
| rs2001945 | 0.04381 | 0.00223 | 1.54E-85 |
| rs174547 | -0.03248 | 0.00235 | 1.61E-43 |
| rs653178 | -0.02137 | 0.00223 | 1.11E-21 |
| rs11856835 | -0.00997 | 0.00223 | 7.54E-06 |
| rs1421085 | -0.01125 | 0.00228 | 7.64E-07 |
| rs58542926 | -0.09007 | 0.00422 | 4.96E-101 |
| rs4420638 | 0.14690 | 0.00284 | 1E-324 |
| rs8121509 | -0.00667 | 0.00224 | 2.95E-03 |
| TRI | | | |
| SNP ID | Estimate | SE | P-value |
| rs1260326 | 0.05411 | 0.00131 | 1E-324 |
| rs1128249 | -0.01941 | 0.00131 | 1.89E-49 |
| rs62260779 | 0.01189 | 0.00215 | 3.22E-08 |
| rs2624847 | 0.00628 | 0.00147 | 1.85E-05 |
| rs1229984 | 0.00636 | 0.00432 | 1.41E-01 |
| rs13107325 | 0.01760 | 0.00244 | 5.89E-13 |
| rs4865796 | -0.00663 | 0.00139 | 1.88E-06 |
| rs1264377 | -0.01066 | 0.00166 | 1.44E-10 |
| rs898137 | -0.00652 | 0.00129 | 4.03E-07 |
| rs13280813 | 0.01131 | 0.00128 | 1.26E-18 |
| rs2001945 | 0.04511 | 0.00128 | 1.66E-269 |
| rs174547 | 0.02571 | 0.00135 | 7.38E-81 |
| rs653178 | 0.00344 | 0.00128 | 7.47E-03 |
| rs11856835 | 0.00343 | 0.00128 | 7.43E-03 |
| rs1421085 | 0.00086 | 0.00131 | 5.12E-01 |
| rs58542926 | -0.05243 | 0.00243 | 1.71E-103 |
| rs4420638 | 0.02716 | 0.00163 | 5.53E-62 |
| rs8121509 | -0.00851 | 0.00129 | 4.13E-11 |
| CRE | | | |
| SNP ID | Estimate | SE | P-value |
| rs1260326 | -0.48653 | 0.04101 | 1.87E-32 |
| rs1128249 | 0.01789 | 0.04099 | 6.63E-01 |
| rs62260779 | 0.15863 | 0.06713 | 1.81E-02 |
| rs2624847 | 0.20305 | 0.04574 | 9.04E-06 |
| rs1229984 | 0.51609 | 0.13500 | 1.32E-04 |
| rs13107325 | 0.14551 | 0.07628 | 5.64E-02 |
| rs4865796 | 0.30590 | 0.04345 | 1.91E-12 |
| rs1264377 | 0.29968 | 0.05187 | 7.58E-09 |
| rs898137 | -0.18879 | 0.04015 | 2.58E-06 |
| rs13280813 | 0.20991 | 0.04007 | 1.62E-07 |
| rs2001945 | -0.36825 | 0.04012 | 4.37E-20 |
| rs174547 | -0.14585 | 0.04213 | 5.37E-04 |
| rs653178 | 0.13566 | 0.04009 | 7.15E-04 |
| rs11856835 | 0.21842 | 0.03997 | 4.66E-08 |
| rs1421085 | 0.01782 | 0.04086 | 6.63E-01 |
| rs58542926 | 0.40100 | 0.07579 | 1.22E-07 |
| rs4420638 | -0.33978 | 0.05101 | 2.72E-11 |
| rs8121509 | -0.16637 | 0.04026 | 3.60E-05 |

**Figure S3.** Tissue-dependent eQTL enrichment Q-Q plots. eQTLs discovered (plotted in red) had significant enrichment for SNPs with pleiotropic effects in at least four metabolic traits, relative to null expectations (in gray) of non-eQTLs. The 6 tissues plotted (out of 48 tissues) were selected based on its extreme enrichment p-values.

**
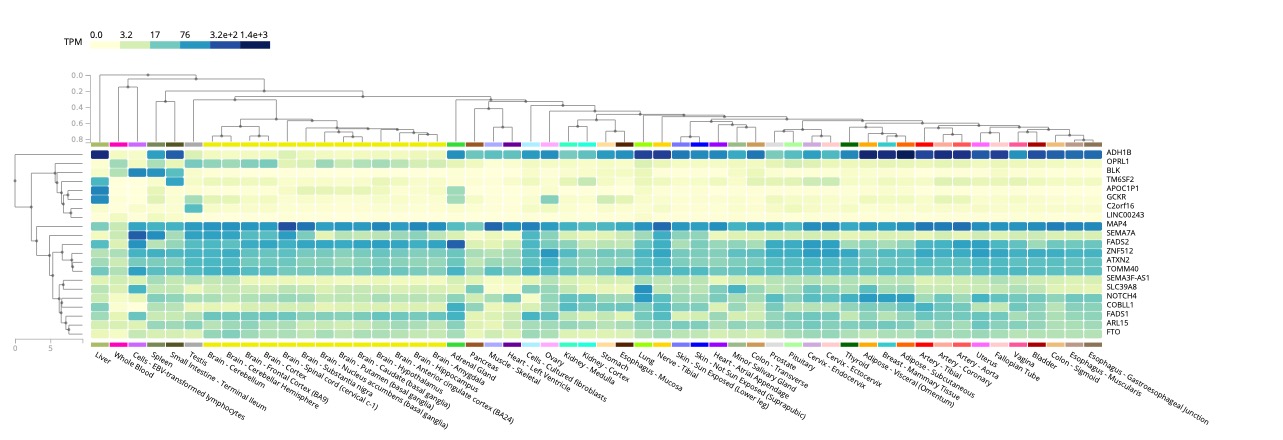
Figure S4.** Heatmap of the average expression (transcripts per million, TPM) of genes found in our study to be associated with more than one MetS-related trait by tissue.
